# Spanish version of the screening Örebro Musculoskeletal Pain Questionnaire: a cross-cultural adaptation and validation

**DOI:** 10.1186/s12955-014-0157-5

**Published:** 2014-10-29

**Authors:** Antonio Ignacio Cuesta-Vargas, Manuel González-Sánchez

**Affiliations:** Departamento de psiquiatría y fisioterapia, Instituto de Investigación Biomédico de Málaga (IBIMA), Universidad de Málaga, C/ Arquitecto Francisco Peñalosa, Ampliación Campus Teatinos, 29071 Malaga, Spain; School of Clinical Sciences, Faculty of Health at the Queensland University of Technology, Queensland, Australia

**Keywords:** Musculoskeletal pain, Reliability, Validity, Outcome, Factor analysis, Clinimetric, Measurement

## Abstract

**Background:**

Spanish is one of the five most spoken languages in the world. There is currently no published Spanish version of the Örebro Musculoskeletal Pain Questionnaire (OMPQ). The aim of the present study is to describe the process of translating the OMPQ into Spanish and to perform an analysis of reliability, internal structure, internal consistency and concurrent criterion-related validity.

**Methods:**

Design: Translation and psychometric testing. Procedure: Two independent translators translated the OMPQ into Spanish. From both translations a consensus version was achieved. A backward translation was made to verify and resolve any semantic or conceptual problems. A total of 104 patients (67 men/37 women) with a mean age of 53.48 (±11.63), suffering from chronic musculoskeletal disorders, twice completed a Spanish version of the OMPQ. Statistical analysis was performed to evaluate the reliability, the internal structure, internal consistency and concurrent criterion-related validity with reference to the gold standard questionnaire SF-12v2.

**Results:**

All variables except “Coping” showed a rate above 0.85 on reliability. The internal structure calculation through exploratory factor analysis indicated that 75.2% of the variance can be explained with six components with an eigenvalue higher than 1 and 52.1% with only three components higher than 10% of variance explained. In the concurrent criterion-related validity, several significant correlations were seen close to 0.6, exceeding that value in the correlation between general health and total value of the OMPQ.

**Conclusions:**

The Spanish version of the screening questionnaire OMPQ can be used to identify Spanish patients with musculoskeletal pain at risk of developing a chronic disability.

**Electronic supplementary material:**

The online version of this article (doi:10.1186/s12955-014-0157-5) contains supplementary material, which is available to authorized users.

## Background

Chronic musculoskeletal conditions have a negative impact, affecting the well-being, independence and physical and psychological health of those who suffer [1]. The incidence of such diseases is very high, with an estimated overall prevalence in the general adult population of a person suffering a low back pain (LBP) episode in his/her lifetime ranging between 60% and 85% [2], with a high impact on socio-economic cost [1,3].

Clinical practice guidelines in primary care based on musculoskeletal evidence show the importance of identifying indicators of poor outcomes need to be considered when achieving the rehabilitative process [4]. Several studies have been published showing the interaction between the patient’s psychosocial status and the probability increase for a musculoskeletal problem becoming chronic [5]. These psychological factors have been shown as good predictors of long-term disabilities [6], developing a correlation in which the experience of pain is associated with disability. Biomedical professionals spend a considerable amount of time with their patients and are aware of the importance that psychosocial factors have. Nevertheless, the ways to face these factors are frequently inappropriate, as is the way of measuring the differences of an eventual intervention [7].

Linton et al. developed the screening questionnaire Örebro Musculoskeletal Pain Questionnaire (OMPQ) in 1998 [8]. This screening questionnaire relates to and assesses the patient’s bio-psychosocial aspects. The scored items evaluate pain location, work absence due to pain, pain duration, pain intensity, control over the pain, frequency of pain episodes in the past three months, functional ability, mood perceptions of work, patient’s estimate of prognosis and fear avoidance [9]. A score of <105 points (max value is 210) indicates low disability, a score between 105 and 130, moderate disability, and a higher score, high disability, where the specificity and sensibility in assessed disability show 0.75 and 0.88 respectively [8]. It has a predictive validity ranging between 0.62–0.75 for pain and 0.68–0.83 for disability [9,10]. It has also shown the same predictability in countries as diverse as Norway and Australia [11]. Additionally, the OMPQ has been externally validated by multiple international studies [10-13]. As a result of all these factors, the OMPQ is currently considered a reference tool when comparing questionnaires of similar characteristics [14].

We have not found published reports indicating that the Spanish versions of the OMPQ have followed the proposed guidelines for translation and cross-cultural adaptation, or that their psychometric properties have been tested. Spanish is one of the five most spoken languages in the world [15]. We created the Spanish version of the screening questionnaire OMPQ to facilitate its introduction and use in Spanish clinical and scientific endeavours.

One of the questionnaires most widely used to evaluate people’s quality of life is the SF-12, whose properties have been demonstrated in more than 400 articles [16]. The main reason for the development of SF-12 was to make a questionnaire that could be reproduced on a single page and completed in a short time (about two minutes), and would be able to represent the summation of the general measures of physical and mental health survey SF-36 with an accuracy level above 90% [17]. By frequency of use and scientific validity, it can be said that the objective was clearly achieved. The main limitations that could be seen in this survey were the number of variables that could be obtained, while the extended version obtained eight items. This problem has been solved with the development SF-12v2 [18], which can obtain the same variables as the SF-36 in less time. The objective of this study is to describe the process of translation of the OMPQ into Spanish and perform analysis of reliability, factor structure, construct validity and concurrent criterion-related validity with the SF-12v2.

## Materials and methods

### Participants

One hundred and four (104) persons participated in the present study. All participants were recruited in a community health centre localized in Torremolinos (Spain). All participants in this study suffer from musculoskeletal disorders (back pain, neck pain or osteoarthritis). The musculoskeletal disorders could be acute, sub-acute or chronic (please, see Table 1 for a more accurate sample description). The inclusion criteria were specific for each of the diseases included. These were: Low back pain: Non-specific low back pain, without radiation to lower limbs, more than six weeks of development [13]; Neck pain: Patients with neck pain and interference in their daily living activities, but no neurological signs; Osteoarthritis: People with joint pain that causes stiffness, deformity and loss of movement.

**Table 1:** **Descriptive data of the sample**

	**Minimum**	**Maximum**	**Mean**	**Stand. Dev**
Age (Years)	29	69	53.48	11.63
Height (cm)	149.0	184.0	164.8	9.09
Weight (Kg)	51	99	74.27	12.08
BMI (Kg/m^2^)	21.5	34.2	27.22	3.51
Pain appearance	Suddenly	**40** (38.5%)		
	Gradually	**64** (61.5%)		
Pain duration	6 w – 3 m	**9** (7.7%)		
	3 m – 12 m	**11** (11.5%)		
	+ 12 m.	**83** (80.5%)		
LBP previous episode	Yes	**95** (92.3%)		
	No	**9** (7.7%)		
Economic compensation	Yes	**9** (7.7%)		
	No	**95** (92.3%)		
Employment status	Active	**64** (61.5%)		
	Sick Leave	**12** (11.5%)		
	Pensioner	**28** (27.0%)		
N (♀/♂)	**104** (67/37)			

By contrast, the exclusion criteria used were common to the three conditions: patient refusal to participate in the study, infectious processes, neoplasm, metastasis, osteoporosis, inflammatory arthritis or fractures and cognitive impairment of any etiologic. Data were collected between April 2013 and December 2013.

### Ethics

All participants, after receiving both written and oral information about the project, signed their informed consent. Ethical approval for the study was granted by the ethics committee of the Faculty of Health Sciences, University of Malaga. This study was conducted in accordance with Ethical Principles for Medical Research Involving Human Subjects (Declaration of Helsinki 2008). The protection of their personal data was performed according to the Spanish Organic Law of Protection of Personal Data 19/55.

## Questionnaires

### The Örebro musculoskeletal pain questionnaire

The Örebro Musculoskeletal Pain Questionnaire [8] is a clinical tool used for identification of patients at risk of developing chronic or long-term musculoskeletal problems. The questionnaire includes 25 questions, of which 21 are rated on a scale of 0–10, with a maximum possible score of 210 points.

The response of the patient will have a value, depending on the question. The value of a response to question 5 will be doubled. For questions 12, 16, 17, 21–25 the score is 10 minus the number that has been circled. For questions 6–11, 13–15, 18–20 the score is the number that has been ticked or circled.

These questions are divided into six different subgroups. The total score is derived from the sum of the values of all items. However, the response to certain questions constrains the value of six different subgroups.

### Short Form 12 Version 2 (SF-12v2)

The SF-12 [17] is composed of a subset of 12 items of the SF-36, selected by multiple regression (selecting one or two items from each of the dimensions of the SF-36), from which, initially, the only ratings were built from a summary of physical and mental components, which, in the second version, became eight components (as in the SF-36). These components are: Physical Function, Physical Role, Emotional Role, Social Function, Mental Health, General Health, Bodily Pain and Vitality [17,18].

## Procedure

### Data collection

Each patient completed the Spanish OMPQ twice, separated by three days, so that any type of treatment would not affect the participant’s situation. On the first occasion, each patient completed the Spanish OMPQ, while on the second, both Spanish OMPQ and SF-12v2 were completed, as different questions allowed a clearer definition of group participants.

### Translation and cultural adaptation of the OMPQ into the Spanish OMPQ

**Figure 1:**
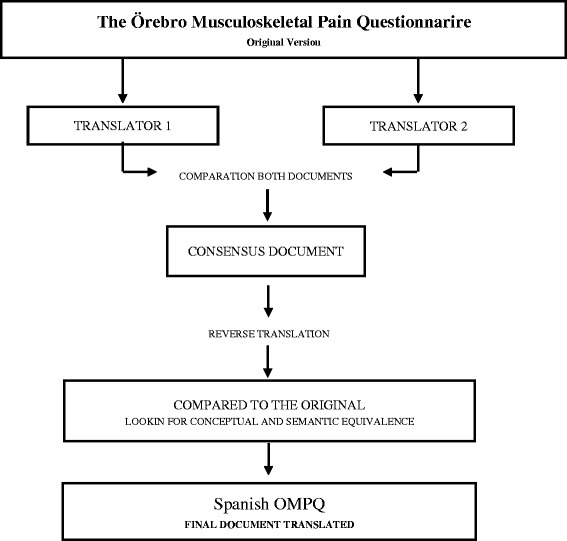
**Flowchart of the development process OMPQ spanish version.**

Following the recommendations of the scientific literature [16], we made a backward translation, so that the equivalence concept used by the author was guaranteed. Here are the steps followed for the translation and back translation of the OMPQ (Figure 1):

1. There were two independent translations, produced by two professional translators, into Spanish. Both versions were compared and, after consensus, drafted into a single document.
2. The original document was compared with a copy of the translation to make a conceptual, as well as semantic, equivalence between the two texts.
3. The document was implemented in clinical practice.

The format of the Spanish adaptation of OMPQ is similar to the original, made with the permission of the author (Additional file 1).

### Statistical analysis

Descriptive analysis was applied to calculate means (±standard deviation) of demographic variables and the Spanish OMPQ. Statistical analysis was performed to evaluate the internal structure, internal consistency and concurrent criterion-related validity. Exploratory factor analysis with maximum likelihood extraction and varimax rotation was estimated for the internal structure of the new questionnaire. Reliability was considered as a test-retest standard deviation of differences either as the 95% limits of agreement [19,20].To determine the internal consistency of the scale items, Cronbach’s α coefficients were calculated. Excellent (ICC >0.80), good (0.80 > ICC >0.60), moderate (0.60 > ICC >0.40) and poor (ICC <0.40) were the levels of reliability [21]. Likewise, concurrent criterion-related validity was taken as the reference questionnaire SF-12v2. Kolmogorov–Smirnov (KS) was performed to determine the distribution of the sample and from this, make a bivariate correlation by Pearson (for parametric) or Spearman (for nonparametric). This analysis was performed using statistical analysis software SPSS version 17.0.

## Results

### Translation and cultural adaptation

Taking the OMPQ original text as a starting point, the two professional translators had virtually identical documents which both facilitated the preparation of the final agreed draft of the document, and resulted in a questionnaire identical to the original with which we started.

The descriptive characteristics of subjects who agreed to complete the questionnaires following the protocol described earlier are shown in Table 1.

### Internal structure: factor analysis

The results obtained in the calculation of the internal structure through factor analysis with maximum likelihood extraction, where the 52.1% of the variance can be explained with three components with an eigenvalue higher than 1 and 10% variance explained and 75.2% with six with an eigenvalue higher than 1, can be observed in Table 2. These six components will be used for the rotated component matrix (Table 3). The rotation process seeks to simplify the structure of looking at how each question saturates the various components in the OMPQ questionnaire decays. Ideally, the saturation level of a question should be close to 1, so that it saturates on a single component. However, it has been noted how some questions have a moderate saturation and saturate on more than one component. Specifically, questions Q11 “Risk Chronic” and Q16 “Belief: not work” saturate on two components. Question Q11 saturates on the components 1 and 4 with levels of 0.462 and 0.569 respectively, while question Q16 saturates on components 1 and 2 with levels of 0.516 and 0.529 respectively. The rest of the saturation levels for each question to saturate the components are shown in Table 3.

**Table 2:** **Total variance explained**

**Component**	**Eigenvalues**		
	**Total**	**% of variance**	**Cumulative%**
1	5.190	24.712	24.712
2	3.566	16.982	41.694
3	2.197	10.461	52.154
4	1.822	8.674	60.829
5	1.659	7.900	68.729
6	1.362	6.486	75.215

Extraction Method: Maximun likehood extraction.

**Table 3:** **Factor structure of rotated component matrix**

	**Component**					
	**1**	**2**	**3**	**4**	**5**	**6**
Q19. Household work	0.919					
Q17. Light work	0.850					
Q18. Walk	0.848					
Q12. Chance working	0.577					
Q20. Shopping	0.522					
Q1.Pain site	0.518					
Q16. Belief: not work	0.516	0.529				
Q11. Risk Chronic	0.462			0.569		
Q7. Frequency		0.700				
Q13. Job satisfaction		0.882				
Q9. Stress			0.880			
Q10. Depressión			0.892			
Q8. Coping				0.692		
Q21. Sleep				0.649		
Q6. Average pain				0.620		
Q3. Duration				0.561		
Q2. Sick leave					0.812	
Q14. Belief: increase					0.707	
Q4. Heavy work						0.851
Q15. Belief: stop						0.500
Q5. Current pain						0.487

Extraction Method: Maximun likehood extraction.

Rotation Method: Varimax with Kaiser Normalization.

Supression at 0.35.

### Reliability and internal consistency

The values obtained after the study on the reliability by calculating the ICC (2, 1), and the corresponding Cronbach α are observable in Table 4. In this table it can be seen how the range of reliability varies between 0.853 (average pain) and 1 (duration) except for the “Coping” variable which has a reliability of 0.218.

**Table 4:** **Reliability and internal consistency**

**Variable**	**Cronbach alpha**	**Lower Bound ICC(2.1) 95%CI**	**Upper Bound ICC(2.1) 95%CI**
Pain site	0.978	0.968	0.985
Sick leave	0.903	0.857	0.934
Duration	1.000	1.000	1.000
Heavy work	0.883	0.828	0.921
Current pain	0.946	0.920	0.963
Average pain	0.853	0.784	0.901
Frequency	0.918	0.879	0.944
Pain	0.936	0.905	0.956
Coping	0.218	−0.153	0.470
Coping	0.218	−0.153	0.470
Stress	0.935	0.904	0.956
Depression	0.861	0.796	0.906
Distress	0.917	0.878	0.944
Risk Chronic	0.939	0.909	0.958
Chance working	0.884	0.829	0.921
Job satisfaction	0.859	0.793	0.905
Return work ex	0.941	0.912	0.960
Belief: increase	0.906	0.861	0.936
Belief: stop	0.879	0.821	0.918
Belief: not work	0.898	0.850	0.931
Fear avoidance	0.928	0.894	0.951
Light work	0.887	0.834	0.923
Walk	0.986	0.980	0.991
Household work	0.892	0.840	0.926
Shopping	0.860	0.793	0.905
Sleep	0.925	0.889	0.949
Function	0.934	0.902	0.955
Total	0.967	0.951	0.978

### Concurrent-criterion validity

Table 5 shows the results obtained by conducting bivariate correlations between variables resulting from the Spanish OMPQ and SF-12 V2. Items that were parameters were calculated through the Pearson correlation coefficient, whereas we used the Spearman correlation coefficient for nonparametric variables. The values of the significant bivariate correlations vary between 0.325 (Physical Role – FA) and 0.610 (General Health –Total). The rest of the bivariate correlations can be seen in Table 5.

**Table 5:** **Bivariate relationships between the different dimensions of OMPQ and SF-12v2**

		**Pain**	**Coping**	**Distress**	**RWE**	**FA**	**Function**	**Total**
**Physical_Function**		**−0.306**	**−0.001**	**0.113**	**−0.103**	−0.405*	−0.460*	−0.427*
	Sig (p)	0.128	0.996	0.581	0.615	0.040	0.018	0.030
***Role_Physical***		**−0.193**	**−0.249**	**0.274**	−0.325*	−0.493**	**−0.263**	−0.366*
	Sig (p)	0.234	0.134	0.086	0.047	0.002	0.100	0.020
**Bodily_Pain**		**0.304**	0.534**	**−0.084**	**0.294**	0.438*	0.573**	0.543**
	Sig (p)	0.132	0.005	0.683	0.145	0.025	0.002	0.004
***General_Health***		0.447**	**0.232**	**0.069**	**0.317**	0.392*	0.542**	0.610***
	Sig (p)	0.006	0.167	0.669	0.056	0.016	0.001	0.000
***Vitality***		**0.077**	**0.120**	**0.292**	**0.209**	**−0.030**	**0.277**	**0.153**
	Sig (p)	0.631	0.465	0.065	0.198	0.848	0.080	0.327
***Social_Functioning***		**−0.161**	−0.367*	−0.344*	**−0.060**	**−0.101**	**−0.240**	**−0.229**
	Sig (p)	0.320	0.027	0.032	0.713	0.529	0.134	0.148
**Role_Emotional**		**−0.151**	−0.585**	**−0.049**	**−0.189**	**−0.121**	**−0.190**	**−0.266**
	Sig (p)	0.462	0.002	0.811	0.354	0.556	0.352	0.189
**Mental_Health**		**−0.080**	**−0.315**	**−0.037**	**−0.098**	**−0.170**	**−0.151**	**−0.200**
	Sig (p)	0.697	0.117	0.859	0.633	0.408	0.463	0.328

*= p<0.05.

**= p<0.005.

***= p <0.001.

## Discussion

The purpose of the present study was to translate and cross-culturally adapt the screening questionnaire OMPQ into Spanish and then to examine the psychometric properties of the Spanish OMPQ. No difficulties were encountered in translating the questionnaire, and the Spanish Version of the OMPQ was demonstrated to be reliable and valid to identify Spanish patients with musculoskeletal pain at risk of developing into a disabling persistent problem.

There are some specialized methods of translation and cross-cultural adaptation of questionnaires of quality of life and health which can greatly facilitate the translation process, especially by avoiding certain problems that may arise in drafting the consensus document, as well as an understanding of reverse translation. Patients demonstrated no problems in completing and understanding the Spanish OMPQ. This facilitated the original author’s approach for the OMPQ.

The determination of a six-factor structure using maximum likelihood extraction was consistent with the previously reported [6,22] and more closely approximate Acute Low Back Pain Screening Questionnaire proposed construct [8,22].

Furthermore, the calculations to assess the concurrent-criterion validity of the Spanish OMPQ, with reference to the general health questionnaire SF-12v2 is possible to observe, as all dimensions demonstrate a significant result with at least one of the variables from the reference questionnaire. Thus, the dimensions “Pain”, “Distress”, and “Work Return Expectancy” show statistically significant correlations with the SF-12v2 variables “General Health”, “Social Functioning” and “Physical Function” respectively. In turn, the dimensions “Coping”, “Function” and “Fear Avoidance” offer significant correlations with a larger number of SF-12v2 variables. The first does so with “Bodily Pain”, “Social Functioning” and “Emotional Role”, moderately significant in the first two correlations. The second of these, however, shows a statistically significant correlation with the variable “Physical Function”, raising the level of significance when correlated moderately with “Bodily Pain and General Health”. However, in the third index, there is a greater number of significant correlations with the variables of SF-12v2. The significance level is moderate when correlated with the variables “Physical Role”, “Physical Function”, “Bodily Pain” and “General Health”. It is important to note that almost all of the correlations that demonstrate a statistical significance have a weak strength of association with the variables of SF-12v2. However, all correlations with indices that are above 0.5 indicate a moderate or high significance. This justifies the conclusion that not only the chance of this relationship is reduced, but also the strength of the correlation between the two questionnaires increases.

The level of confidence shown by the Spanish OMPQ is excellent as rated by Mancini et al. [21]. All levels of reliability were shown superior to 0.85 except for “Coping”, which had a poor reliability displaying a value of 0.218.These values demonstrated the high stability of all the items in the Spanish OMPQ.

A weakness of this study is that no outcome tool is used to quantify change over time as a correlation to time and recovery, so there are no cut offs that are specific to a Spanish population. Hence, in terms of true validity, the tool can only be used in those populations where the original tool is validated but they have Spanish speakers as well. The lack of predictive capacity in the Spanish version of the OMPQ indicates that it is recommendable to use the predictive ability of other versions [8,17,23,24]. Future work needs to validate the predictive capacity in Spanish speaking populations and this cannot be done till the scale is presented in a translated form which this study did. In addition, the sample included acute, sub-acute and chronic musculoskeletal disease patients and the psychometric properties of the questionnaire may be different among acute and chronic pain patients.

Analyzing the results, the main conclusion of this study is that the Spanish version of the screening questionnaire OMPQ is a tool that can be used as an instrument with a high reliability for assessment and monitoring of patients with musculoskeletal pain at risk of a chronic disability.

## Additional file

Additional file 1:
**Örebro Musculoskeletal Pain Questionnaire Spanish Version.**

## Abbreviations

KS: Kolmogorov – smirnov
LBP: Low back pain
OMPQ: Örebro musculoskeletal pain questionnaire
