# Supplementary material for: Spanish version of the screening Örebro Musculoskeletal Pain Questionnaire: a cross-cultural adaptation and validation
Source: Health Qual Life Outcomes. 2014 Oct 29;12:157. doi: 10.1186/s12955-014-0157-5 (PMC4219048; doi:10.1186/s12955-014-0157-5)
Supplement: Additional file 1: — Örebro Musculoskeletal Pain Questionnaire Spanish Version. [file 12955_2014_157_MOESM1_ESM.pdf]

Additional file 1

ÖREBRO MUSCULOSKELETAL PAIN QUESTIONNAIRE

Spanish Version

---

1. **Nombre:**
   Teléfono: **Fecha:**
2. **Fecha de la lesión:** **Fecha de nacimiento:**
3. **Sexo:**
4. **Dónde nació:**

Estas preguntas y afirmaciones son aplicables si tiene molestias o dolores como en el cuello, la espalda, el hombro.
Por favor lea y responda las preguntas cuidadosamente. No tome demasiado tiempo para responder a las preguntas, sin embargo, es importante que las conteste todas. Siempre encontrará una respuesta para su situación particular.

5. **¿Dónde le duele? Marque la(s) casilla(s) correspondiente(s)**

☐ Cuello ☐ Hombro ☐ Brazo ☐ Parte alta de la espalda

☐ Parte Baja ☐ Pierna ☐ Otro (Indicar): ___________________

2 x (Max 10) ____

6. **¿Cuántos días de trabajo ha perdido a causa del dolor durante los últimos 18 meses? Marque sólo una.**

☐ 0 Días (1) ☐ 1 - 2 Días (2) ☐ 3 - 7 Días (3) ☐ 8 - 14 Días (4)

☐ 15 - 30 Días (5) ☐ 1 Mes (6) ☐ 2 Mes (7)

☐ 3 – 6 Meses (8) ☐ 6 – 12 Meses (9) ☐ Más de un año (10)

____

7. **¿Desde hace cuanto tiempo sufre su dolor actual? Marque sólo una.**

☐ 0 Días (1) ☐ 1 - 2 Días (2) ☐ 3 - 7 Días (3) ☐ 8 - 14 Días (4)

☐ 15 - 30 Días (5) ☐ 1 Mes (6) ☐ 2 Mes (7)

☐ 3 – 6 Meses (8) ☐ 6 – 12 Meses (9) ☐ Más de un año (10)

____

8. **¿Es su trabajo pesado o monótono? Marque la mejor opción.**

0 1 2 3 4 5 6 7 8 9 10

No en absoluto Extremadamente

____

9. ¿Cómo calificaría usted el dolor que tuvo durante la semana pasada? Marque una.

0 1 2 3 4 5 6 7 8 9 10

No dolor — Peor dolor soportable.

10. En los últimos tres meses, de media, ¿Cómo de malo fue su dolor en una escala de 0 – 10? Marque uno.

0 1 2 3 4 5 6 7 8 9 10

No dolor — Peor dolor soportable.

11. ¿Con qué frecuencia diría usted que ha experimentado episodios de dolor, de media, durante los últimos tres meses? Marque uno

0 1 2 3 4 5 6 7 8 9 10

Nunca — Siempre.

12. Sobre la base de todas las cosas para hacer frente, o lidiar con su dolor, durante un día normal ¿Cuánto es capaz de disminuirlo? Marque uno.

10 - x

0 1 2 3 4 5 6 7 8 9 10

No consigo disminuirlo — Consigo eliminarlo por c[illegible]

13. ¿Cómo de tenso(a) o ansioso(a) se ha sentido durante la última semana? Marque uno.

0 1 2 3 4 5 6 7 8 9 10

Absolutamente calmado y relajado — Tenso y nervioso como nunca

14. ¿Cuánto se ha sentido molesto(a), por una sensación depresiva, durante la última semana? Marque uno.

0 1 2 3 4 5 6 7 8 9 10

Nunca — Extremadamente

15. En su opinión, ¿Cómo de grande es el riesgo de que su dolor actual puede llegar a ser persistente? Marque uno

0 1 2 3 4 5 6 7 8 9 10

Ningún riesgo — Riesgo muy alto

16. En su opinión, ¿Cuáles son las probabilidades de que usted será capaz de trabajar en seis meses?

10 - x

0 1 2 3 4 5 6 7 8 9 10

Ninguna probabilidad — Muchas posibilidades

17. Considerando sus rutinas de trabajo, gestión, salario, posibilidades de promoción y compañeros de trabajo, ¿Cual es su grado de satisfacción con su trabajo?

10 - x ______

0 1 2 3 4 5 6 7 8 9 10

Absolutamente insatisfecho — Completamente satisfecho

Éstos son algunas de las cosas que otras personas nos han dicho acerca de su dolor. Para cada afirmacion, marque un número de 0 a 10 para decir cuánto afectaría a su dolor las actividades físicas, tales como agacharse, levantar objetos, caminar o conducir.

---

18. La actividad física hace que mi dolor empeore.

______

0 1 2 3 4 5 6 7 8 9 10

Completamente en <u>des**acuerdo**</u> — Completamente de <u>**acuerdo**</u>

19. Un aumento del dolor es una indicación de que debo dejar lo que estoy haciendo hasta que disminuye o desaparece.

______

0 1 2 3 4 5 6 7 8 9 10

Completamente en <u>des**acuerdo**</u> — Completamente de <u>**acuerdo**</u>

20. No debería realizar mi trabajo habitual con mi dolor actual.

______

0 1 2 3 4 5 6 7 8 9 10

Completamente en <u>des**acuerdo**</u> — Completamente de <u>**acuerdo**</u>

Le indicamos una lista con cinco actividades. Marque el número que mejor describe su capacidad actual de participar en cada una de estas actividades.

---

21. Puedo realizar una actividad ligera durante una hora.

10 - x ______

0 1 2 3 4 5 6 7 8 9 10

No puedo hacerlo debido a mi Dolor — El dolor no es un problema

22. Puedo caminar durante una hora.

0	1	2	3	4	5	6	7	8	9	10	10 - x

No puedo hacerlo debido a mi Dolor El dolor no es un problema

23. Puedo hacer las tareas domésticas.

0	1	2	3	4	5	6	7	8	9	10	10 - x

No puedo hacerlo debido a mi Dolor El dolor no es un problema

24. Puedo hacer la compra semanal.

0	1	2	3	4	5	6	7	8	9	10	10 - x

No puedo hacerlo debido a mi Dolor El dolor no es un problema

25. Puedo dormir por la noche.

0	1	2	3	4	5	6	7	8	9	10	10 - x

No puedo hacerlo debido a mi Dolor El dolor no es un problema
